# Assessment of knowledge, attitudes, and practices regarding microbiota composition and influencing factors among the general population in Jazan province: A cross-sectional study

**DOI:** 10.5455/javar.2023.j733

**Published:** 2023-12-31

**Authors:** Bander S. Rajab, Riyadh A. Jahlan, Ameer M. Mobarki, Osama A. Alhazmi, Ehab F. Hakami, Waleed H. Shayari, Nawaf A. Darabshi, Abdulgader K. Basamad, Abdulaziz H. Alhazmi

**Affiliations:** Faculty of Medicine, Jazan University, Jazan, Saudi Arabia

**Keywords:** Gut microbiota, probiotics, knowledge, attitudes, practices, Jazan, Saudi Arabia

## Abstract

**Objective:**

The human gut microbiota is crucial to maintaining health and preventing diseases. The general population‘s knowledge, attitudes, and practices regarding microbiota composition and the factors affecting it remain poorly understood in Saudi Arabia. The present cross-sectional study aimed to evaluate the level of knowledge, attitudes, and practices among the general population in the Jazan Province of Saudi Arabia regarding the gut microbiota and its main influencing factors.

**Materials and Methods:**

A descriptive, cross-sectional design was employed, utilizing a validated self-administered online questionnaire to collect data from participants aged 18 years and older. The study population excluded nonresidents of the Jazan region, individuals below 18, and those who declined to participate. Nonrandomized convenience sampling was used to recruit participants from the Jazan Province, targeting the general population.

**Results:**

One thousand one hundred twenty-six participants completed the survey, resulting in a response rate of 93%. Most participants (50.4%) had average microbiota knowledge, 14.7% demonstrated good understanding, and 34.9% had poor knowledge. Regarding probiotics, the findings indicated that 76.4% of participants exhibited insufficient knowledge, 21.1% had average knowledge, and 2.5% displayed good knowledge. Most participants (65.7%) held a neutral attitude toward antibiotics and probiotics.

**Conclusion:**

This study highlights a significant gap in understanding probiotics among the general population in the Jazan Region. Comprehensive education and awareness campaigns are urgently needed to promote a better understanding of microbiota composition, its significance for health, and the potential benefits of probiotics. Effective public health initiatives should be developed to provide accurate and up-to-date probiotic information, fostering positive health outcomes.

## Introduction

The term “microbiota” refers to the entire population of microbes that inhabit a specific area. This group of microbes includes bacteria, fungi, archaea, viruses, and protozoa [1]. The microbiota in the human body protects against different illnesses by serving as a physical barrier against pathogens, stopping colonization by consuming the available nutrients, and generating antimicrobial substances. The gut microbiota refers to a group of microorganisms, primarily bacteria, that inhabit the human gastrointestinal tract (GIT). These microorganisms are estimated to number between 10^13^ and 10^14^ and weigh about 2 kg, surpassing the total number of cells in the human body [2]. It predominantly consists of anaerobic microorganisms and plays critical roles in metabolic, physiological, and immunological processes. For instance, certain microbiotas such as bacteroidetes and firmicutes are primarily found in the lumen of the proximal part of the colon and contribute to nutrient absorption through the fermentation of polysaccharides into short-chain fatty acids; most of these microorganisms are anaerobic [3].

Probiotics are an external source of beneficial bacteria that help to maintain and restore the average intestinal microbiota balance [4]. Probiotics are described as “live microorganisms that, when administered in adequate amounts, confer a health benefit on the host” by the World Health Organization [5]. There are foods, drinks, and medications that contain probiotics. In addition, probiotic products include various beneficial bacteria, such as *Lactobacillus* and *Bifidobacterium*, that can colonize the GIT and achieve a better balance of microflora [6]. The gut microbiome has many vital functions in the human body, including metabolic, physiological, and immunological processes. The significant impact of it is on the metabolic process, which has been shown to help absorb nutrients [7]. The composition and function of the microbiota could be affected by certain factors, such as diet, diseases, and antibiotic use. Changes in diets can result in a significant difference in the microbial population as early as 24 h. Hence, dietary intervention is an attractive option for altering the gut microbiota [7,8]. Moreover, the gut microbiota and its diversity can be adversely affected by higher stress levels resulting from contemporary lifestyles, potentially leading to an increased prevalence of *Clostridium* [2].

Multiple environmental factors impact the gut microbiome, possibly contributing to the development of some diseases [7,8]. For example, patients diagnosed with inflammatory bowel disease (IBD) have less bacterial diversity, resulting in an imbalance between normal and harmful bacteria. To overcome this situation, patients with IBD show improvement when taking probiotics [7,8]. Using broad-spectrum antibiotics could result in bacterial resistance, dysbiosis, drops in diversity, and altered microbiome compositions. Antibiotics also affect protein activity, gene expression, and metabolism within the gut microbiota. Notably, the overuse of antibiotics increases the risk of intestinal infections, including those caused by new microbes or the overgrowth of opportunistic organisms already in the intestine. *Clostridium difficile* is commonly associated with antibiotic-associated diarrhea and potentially fatal pseudomembranous colitis. After cessation of antibiotic treatment, the microbiota may partially or fully recover its original composition, depending on the antibiotic class, dosage, and duration of exposure [7,8]. The most crucial concern with antibiotics is the increased risk of intestinal infections, possibly from new microbes or the overgrowth of opportunistic organisms already in the intestine [9,10]. Despite this, certain factors can positively impact the gut microbiota. One such factor is exercise, which increases microbial diversity and encourages the growth of bacteria that produce butyrate, a compound known for its health-enhancing and anti-inflammatory properties [2].

Given the limited research conducted on the knowledge, attitudes, and practices of the general population in Saudi Arabia regarding gut microbiota and its influencing factors, there is a need to assess these aspects. Understanding the general population‘s knowledge, attitudes, and practices regarding gut microbiota is crucial due to its significant role in maintaining overall well-being and designing interventions that target factors to improve the general understanding of gut microbiota. This study aimed to comprehensively assess the knowledge, attitudes, and practices of the general population in Jazan Province, southwestern Saudi Arabia, regarding gut microbiota and the primary influencing factors influencing them, thereby identifying critical knowledge gaps in this domain. By identifying these factors, this study contributes to improving our understanding of gut microbiota and informs interventions to maintain healthy microbiota and prevent associated health problems.

## Materials and Methods

### Ethical consideration

The study was conducted per ethical principles and was approved by the Jazan University Scientific Research Ethics Committee (reference number REC-44/09/597). All participants were provided information about the study objectives and their right to decline participation or withdraw at any time. In addition, participants were assured of their data’s anonymity and full confidentiality. All authors confirm that this study complies with the Declaration of Helsinki.

### Study design and target population

A descriptive, cross-sectional study was conducted to assess the knowledge, attitudes, and practices of the Jazan Province‘s general population regarding the microbiota‘s role and the main factors affecting it, including probiotics and antibiotics. Jazan is one of the 13 provinces in Saudi Arabia, located on the country‘s southwestern border, with a population of over 1.4 million residents, according to the 2022 census done by the Saudi General Authority of Statistics. The study population comprised adult residents of the Jazan Province who were 18 or older and spoke Arabic. Participants who refused to participate, nonresidents of the Jazan Province, and those under 18 years of age or who did not speak Arabic were excluded from the study. Nonrandomized convenience sampling was used to recruit participants from the 17 regions in the Jazan Province, targeting the general population. We determined the required sample size using Raosoft software from the website http://www.raosoft.com/samplesize.html, considering a 95% confidence level, an estimated response distribution of 50%, and a margin of error of ±3%. Raosoft software (http://www.raosoft.com/samplesize.html) calculated the minimum required sample size to be 1,212 participants.

### Data collection tool

A validated, self-administered online questionnaire was used to collect the data. It was based on a previous study that looked at the knowledge, attitudes, and practices of the people in the United Arab Emirates (UAE) about gut microbiota and the main things that affect it [11]. The questionnaire consisted of 43 questions, which were divided into four sections. The first section comprised seven questions related to demographic information, including sex, age, educational degree, occupation, area of residence, social status, and socioeconomic status. The second section aimed to evaluate participants‘ knowledge of the microbiota and consisted of 12 questions. The third and fourth sections of the questionnaire assessed participants‘ attitudes and practices toward the main factors affecting the microbiota and contained 24 questions. The questionnaire included multiple-choice, true/false questions, and five-item Likert scales. To ensure the validity and reliability of the adapted questionnaire, a pilot study was conducted on 30 participants, and they were subsequently excluded from the final analysis. The internal reliability of the questionnaire was assessed using Cronbach‘s alpha, which was 0.788.

### Data collection process and data analysis

We collected data between April 2023 and June 2023 by administering an online survey through Google Forms. The questionnaire was distributed through various social networking applications, including WhatsApp, Twitter, Instagram, Facebook, and Telegram. We provided the participants with information regarding the purpose of the study and made them aware of their right to voluntarily exit the study at any given moment. After collecting the data, we manually verified and coded it within an Excel sheet before analyzing it using Statistical Product and Service Solutions version 26. We analyzed the data using descriptive and comparative statistics. We calculated descriptive statistics for study variables, including frequency and percentage for qualitative variables and mean and standard deviation for quantitative variables. We used the chi-square test to identify any sociodemographic factors associated with levels of knowledge, attitudes, and practices toward microbiota, determining statistical significance with a *p*-value of less than 0.05. Stepwise multiple linear regression (MLR) was used to identify significant microbiota and probiotic knowledge predictors. Correct answers to the knowledge questions scored one point to evaluate participants‘ knowledge of microbiota and probiotics, while incorrect answers received no points. A scoring system was developed based on the total points achieved, with poor knowledge defined as 0–3 points, average knowledge defined as 3–6 points, and good knowledge defined as 7–9 points.

Participants‘ attitudes and practices toward the main factors affecting microbiota were assessed using three-item Likert scales, with a point assigned for affirming a true statement or disagreeing with a false one. The total score was calculated based on the points achieved, and a scoring system was developed to categorize participants‘ attitudes and practices as negative (9–15 points), neutral (16–21 points), or good (22–27 points).

## Results

Table 1 represents the demographic distribution of the participants. A survey was completed by 1,126 participants from the Jazan region of Saudi Arabia, with a response rate of 93%. Most participants were male (53.8%) and aged between 18 and 29 years (63.1%). Most participants had a university degree (72.6%) and were university students (51.4%). Regarding monthly family income, the majority of study participants had a monthly family income of less than 10,000 Saudi riyals (SR), with 24.2% reporting an income of less than 5,000 SR and 25.0% reporting an income between 5,001 and 10,000 SR. A significant proportion of participants (20.6%) reported a monthly income between 10,001 and 15,000 SR, while a smaller proportion reported higher incomes. Only 13.0% reported an income of more than 20,000 SR per month.

**Table 1.** Demographic distribution of the participants (*n =* 1126).

Demographics		*N*	%
Gender	Male	606	53.8%
	Female	520	46.2%
Age (groups)	18–29	710	63.1%
	30–39	170	15.1%
	40–49	165	14.7%
	50 and above	81	7.2%
Educational level	High School or below	254	22.6%
	University graduate	817	72.6%
	Postgraduate degree	55	4.9%
Occupation	University Student	579	51.4%
	Healthcare worker	52	4.6%
	Nonhealthcare worker	300	26.6%
	Retired	42	3.7%
	Looking for a job	111	9.9%
	Freelancer	42	3.7%
Average monthly family income (SR)	Less than 5000 SR	273	24.2%
	5,001–10,000 SR	281	25.0%
	10,001–15,000 SR	232	20.6%
	15,001–20,000 SR	194	17.2%
	More than 20,000 SR	146	13.0%
	Total	1126	100%

**Figure 1.**
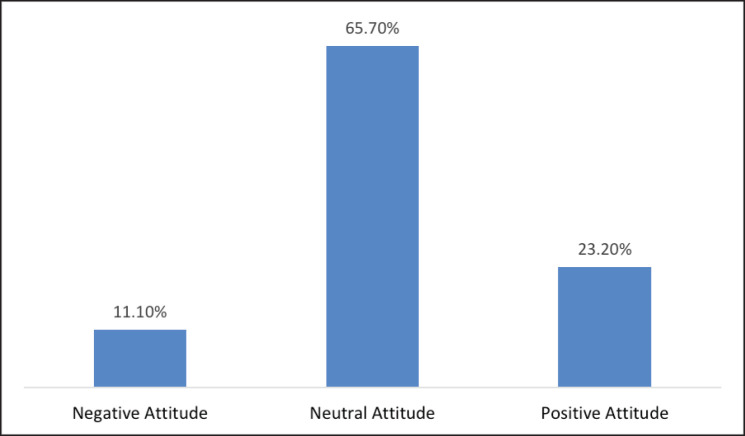
Attitudes of the participants regarding antibiotics and probiotics.

Table 2 represents participants‘ knowledge regarding microbiota and probiotics. The results showed that 79.8% (*n =* 898) of the participants correctly defined microbiota as “living bacteria inside a human body.” Most participants (50.4%) had average knowledge of the microbiota, while 14.7% had good knowledge and 34.9% had poor knowledge. For probiotics, the results showed that 76.4% of the participants had poor knowledge about probiotics, 21.1% had average knowledge, and only 2.5% had good knowledge. The study also assessed the attitudes of participants toward antibiotics and probiotics, as illustrated in Figure 1. The results showed that most participants (65.7%) had a neutral attitude toward antibiotics and probiotics, while 23.2% had a positive attitude and 11.1% had a negative attitude.

**Figure 2.**
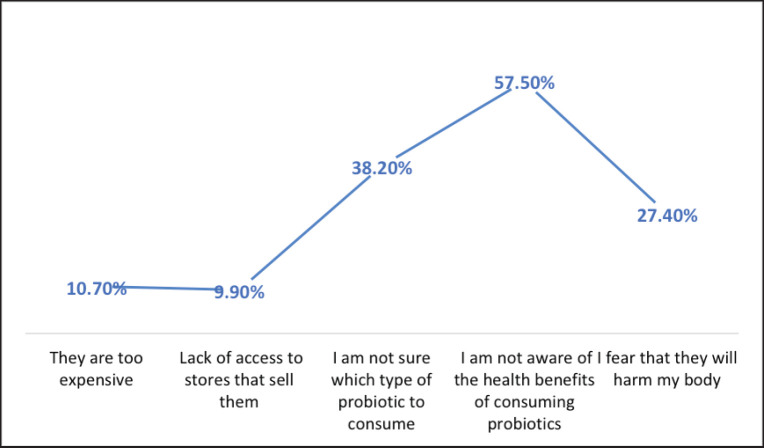
General barriers against probiotics as stated by the participants.

Figure 2 represents the general barriers against probiotics, as stated by the participants. The most commonly reported barrier was the lack of awareness of the health benefits of consuming probiotics, with 57.5% of the participants reporting this as a barrier. The second most commonly reported barrier was uncertainty about which type of probiotic to consume, with 38.2% of the participants reporting this as a barrier. Other reported barriers included a fear that probiotics will harm the body (27.4%), probiotics being too expensive (10.7%), and a lack of access to stores that sell probiotics (9.9%). Table 3 represents the knowledge of microbiota across different demographic groups. The total knowledge score in various groups was analyzed to determine the factors associated with better understanding. Regarding the average monthly family incomes, higher-income participants reported better knowledge than the others (*p* < 0.001). According to the gender difference, most of both genders reported average knowledge, and 17.8% (*n =* 108) of males reported higher good knowledge of microbiota in comparison to females 11.0% (*n =* 57) (*p* < 0.002). Regarding occupation, there was a significant difference in the occupation type and microbiota knowledge (*p* < 0.021). Healthcare workers, 19.2% (*n =* 10), followed by university students, 17.6% (*n =* 102), reported a higher prevalence than others with different occupations. According to educational level, participants with postgraduate degrees reported the highest level of good knowledge at 20.0% (*n =* 11), followed by university graduates at 14.6% (*n =* 119), and participants with high school degrees or below had the lowest average of good knowledge at 13.8% (*n =* 35) (*p* < 0.026).

**Table 2.** Knowledge of the participants toward microbiota and probiotics.

Parameters	Knowledge toward microbiota		Knowledge toward probiotics	
	*N*	%	*N*	%
Poor knowledge	393	34.9%	860	76.4%
Average knowledge	568	50.4%	238	21.1%
Good knowledge	165	14.7%	28	2.5%

Table 4 represents the multiple logistic regression analysis of only variables significantly associated with higher knowledge; in the multiple logistic regression analysis, after adjusting for other variables, males were more significantly associated with poor knowledge than females (*p =* 0.001 OR = 0.516). Males also had more average knowledge than females (*p =* 0.006 and OR = 0.583). Regarding the monthly incomes, participants with less than 10,000 SR income showed poorer understanding than those with monthly incomes above 20,000 SR (*p* = 0.0001 and OR = 3.327–3.713). In addition, the MLR model showed that those with monthly incomes less than 10,000 SR were 2.5 times more likely to have average knowledge than the others with 20,000 SR monthly incomes (*p* = 0.0001–0.006 and OR = 2.202–2.877).

## Discussion

The study, conducted in the Jazan region of Saudi Arabia, aimed to assess the knowledge and attitudes of participants toward microbiota and probiotics. The study surveyed 1,126 participants, most of whom were male (53.8%) and aged between 18 and 29 (63.1%). Most participants had a university degree (72.6%) and were university students (51.4%). Regarding microbiota, the study found that while 79.8% of participants correctly defined microbiota as “living bacteria inside a human body,” only 14.7% had good knowledge about microbiota. The majority (50.4%) had an average level of knowledge, and 34.9% had poor knowledge. The study found that 76.4% of participants had poor knowledge about probiotics, while only 2.5% had good knowledge. The study also assessed participants‘ attitudes toward antibiotics and probiotics and found that the majority (65.7%) had a neutral attitude toward antibiotics and probiotics. In comparison, 23.2% had a positive attitude, and 11.1% had a negative attitude. This suggests a relatively low level of awareness about the potential benefits of probiotics and the risks associated with the overuse of antibiotics among the study population. Furthermore, most participants could define microbiota as “living bacteria inside a human body,” but their knowledge regarding its role in protecting against disease and boosting immunity was lacking. The study also revealed misconceptions among participants regarding the presence of microorganisms in the body, with more than half believing that having bacteria in the nose is dangerous and that microorganisms in the intestines could cause diseases such as diarrhea. This highlights the need for increased education and awareness-raising efforts to correct these misconceptions. The study also found that age played a significant role in shaping people‘s knowledge on the topic, with younger individuals having more excellent knowledge compared to those in older age groups, possibly due to higher exposure to the internet and social media as sources of information on health-related topics. In terms of education, university students had better knowledge regarding microbiota than other educational groups, which is especially important since microbiota concerns healthcare professionals (HCPs) and medical students. While the study found that HCPs had better knowledge than nonmedical personnel, the difference was insignificant, suggesting that there is still room for improvement in the knowledge of HCPs regarding microbiota.

The majority of our participants did not demonstrate adequate knowledge of probiotics. With only 2.5% of the participants showing a good understanding of probiotics, the overwhelming majority (76.4%) demonstrated poor knowledge. This shows a significant lack of awareness of probiotics among the public. A study conducted in Al Qassim, Saudi Arabia, revealed that nearly 73% of the respondents were unfamiliar with the term probiotics [12]. In another study carried out in the UAE, only 4% of the participants showed an adequate understanding of probiotics, with variances depending on the existence of a medical background. Compared to nonmedical respondents, HCPs were almost ten times more likely to have good and adequate knowledge of probiotics [11]. The international figures seem similar to those reported in the region. For example, in a study involving hospitalized patients in Chicago, where 44% of respondents were aware of probiotics, only 20% could choose the correct definition [13]. In contrast, a study assessing the knowledge of HCPs conducted in Nigeria revealed that roughly 72% of respondents were aware of probiotics [14]. 88.7% of the college students surveyed in India knew what probiotics were made of [15]. In addition, a study of college students in the Philippines found that participants had a high level of probiotic knowledge and consumption [16]. HCPs, due to their medical backgrounds, are expected to possess greater knowledge about probiotics compared to the general population [11,14]. When compared to HCPs and college students, there is a clear knowledge gap between the public and these groups. Nevertheless, our study‘s findings suggest a lack of awareness and understanding of probiotics among the general population. It is evident from these findings that public awareness and understanding of probiotics are notably deficient, emphasizing the pressing need for targeted educational interventions to bridge the knowledge gap. Efforts to enhance public knowledge about probiotics can play a pivotal role in promoting healthier lifestyles and empowering individuals to make informed choices regarding their gut health.

**Table 3.** Factors associated with different levels of knowledge of microbiota.

Parameters		Knowledge toward microbiota						*p*-value
		Poor knowledge		Average knowledge		Good knowledge		
		*N*	%	*N*	%	*N*	%	
Gender	Male	193	31.8%	305	50.3%	108	17.8%	0.002*
	Female	200	38.5%	263	50.6%	57	11.0%	
Age (groups)	18–29	241	33.9%	351	49.4%	118	16.6%	0.135
	30–39	66	38.8%	87	51.2%	17	10.0%	
	40–49	63	38.2%	81	49.1%	21	12.7%	
	50 and above	23	28.4%	49	60.5%	9	11.1%	
Educational level	High school or below	109	42.9%	110	43.3%	35	13.8%	0.026*
	University graduate	269	32.9%	429	52.5%	119	14.6%	
	Postgraduate degree	15	27.3%	29	52.7%	11	20.0%	
Occupation	University student	188	32.5%	289	49.9%	102	17.6%	0.021*
	Healthcare worker	15	28.8%	27	51.9%	10	19.2%	
	Nonhealthcare worker	105	35.0%	159	53.0%	36	12.0%	
	Retired	16	38.1%	23	54.8%	3	7.1%	
	Looking for a job	53	47.7%	46	41.4%	12	10.8%	
	Freelancer	16	38.1%	24	57.1%	2	4.8%	
Average Monthly Family Income (SR)	Less than 5,000 SR	116	42.5%	123	45.1%	34	12.5%	<0.0001*
	5,001–10,000 SR	115	40.9%	141	50.2%	25	8.9%	
	10,001–15,000 SR	76	32.8%	122	52.6%	34	14.7%	
	15,001–20,000 SR	48	24.7%	115	59.3%	31	16.0%	
	More than 20,000 SR	38	26.0%	67	45.9%	41	28.1%	

SR: Saudi riyals.*p-values less than 0.05 are considered statistically significant.

Only 11.1% of those who participated in the current study displayed a negative attitude, showing they would not consider probiotics and were unwilling to learn about them. In comparison, 23.3% showed a positive attitude. However, the vast majority of participants exhibited a neutral perspective. These findings were similar to those of a study in the UAE that revealed none of the 450 participants had a positive attitude toward probiotics, with the majority displaying neutral or negative attitudes instead [11]. In Australia, Khalesi et al. [17] found that more than 40% of the participants showed negative attitudes and behaviors toward probiotics [17]. Our participants‘ neutral and negative attitudes could be due to a lack of awareness and knowledge of the health benefits that probiotics can bring. This showed, especially when asked about the barriers that kept them from using probiotics. The most popular answer was that they were unaware of any positive health effects, and they were also clueless about which type of probiotic was more suitable for their use, which suggests a lack of information about probiotics. The prevalence of neutral and negative attitudes toward probiotics among the participants in our study and other studies [11,17] underscores the pressing need for targeted educational interventions. Understanding the specific barriers that hinder probiotic usage, such as the lack of awareness regarding their health benefits and the suitable probiotic strains, can guide the development of effective educational campaigns. By addressing these knowledge gaps and misconceptions, we can empower individuals to make informed decisions and embrace the potential benefits of probiotics for their gut health and overall well-being.

**Table 4.** Multiple logistic regression of the factors associated with higher knowledge of microbiota.

Knowledge of microbiota^a^		*p*-value	OR ^	95% confidence interval for OR ^	
				Lower	Upper
Poor knowledge	Male	0.001*	0.516	0.344	0.773
	Female (ref.)				
	High school or below	0.190	1.898	0.727	4.954
	University graduate	0.397	1.468	0.604	3.567
	Postgraduate degree (ref.)				
	University student	0.102	0.273	0.058	1.296
	Healthcare worker	0.157	0.289	0.052	1.611
	Nonhealthcare worker	0.456	0.552	0.115	2.637
	Retired	0.950	0.935	0.116	7.530
	Looking for a job	0.391	0.490	0.096	2.498
	Freelancer (ref.)				
	Less than 5,000 SR	0.0001*	3.327	1.823	6.072
	5,001–10,000 SR	0.0001*	3.713	1.963	7.022
	10,001–15,000 SR	0.043*	1.912	1.021	3.582
	15,001–20,000 SR	0.211	1.517	0.790	2.915
	More than 20,000 SR (ref.)				
Average knowledge	Male	0.006*	0.583	0.397	0.856
	Female (ref.)				
	High school or below	0.643	1.231	0.510	2.970
	University graduate	0.370	1.444	0.647	3.219
	Postgraduate degree (ref.)				
	University student	0.074	0.250	0.055	1.141
	Healthcare worker	0.107	0.257	0.049	1.341
	Nonhealthcare worker	0.247	0.406	0.088	1.868
	Retired	0.704	0.676	0.090	5.101
	Looking for a job	0.113	0.274	0.055	1.357
	Freelancer (ref.)				
	Less than 5,000 SR	0.006	2.202	1.259	3.850
	5,001–10,000 SR	0.000	2.877	1.588	5.212
	10,001–15,000 SR	0.035	1.844	1.043	3.262
	15,001–20,000 SR	0.011	2.100	1.181	3.734
	More than 20,000 SR (ref.)				

SR: Saudi riyals. ^a^Good-knowledge is the reference. *p-values less than 0.05 are considered statistically significant.

As stated in the literature, the use of antibiotics is a significant contributor to dysbiosis. Regarding antibiotic use, almost one-third (27.3%) of the study population had used antibiotics without a medical prescription, similar to the result of a previous study conducted in the region in 2021, in which 25% used antibiotics without a prescription. These percentages seem not far from what was reported in the Western region of Saudi Arabia (26.1%) and Kuwait (27.5%) [18–20]. However, this value was higher than the rates in Hong Kong, the UK, Malaysia, European countries, and Indonesia, which ranged between 4.8% and 9% [21–25]. Over 40% of the participants in our study reported that they discontinued antibiotics once they felt better rather than completing the entire course of therapy. This behavior can contribute to developing bacterial resistance, which can be challenging to treat. In the study conducted in the western region of Saudi Arabia, over 50% of the participants stopped taking antibiotics after they felt improvement [19]. Conversely, in a Romanian study, only 10% of participants discontinued antibiotic use once they felt better, while approximately 60% continued taking them until the doctor-recommended course was completed [26]. Furthermore, one-third of the participants in our study reported that they would recommend antibiotics to friends and family or use them as a precaution. A survey conducted in the UAE also found similar results, with 34% of nonmedical participants preferring to advise family and friends to take their antibiotics for similar diseases, while 31% chose to use antibiotics as a precaution [11]. The prevalence of self-medication with antibiotics and the tendency to discontinue the treatment prematurely among a substantial proportion of participants in our study underscore the urgent need for public awareness campaigns about the responsible use of antibiotics. These campaigns should emphasize the potential consequences of self-prescribing antibiotics without medical supervision, such as the development of antibiotic resistance, which poses a significant challenge in the management of bacterial infections. By promoting appropriate antibiotic use and adherence to prescribed treatment courses, we can collectively work toward preserving the effectiveness of antibiotics and safeguarding public health from the threat of antimicrobial resistance.

This study has several strengths. First, this study offers insightful information on the knowledge, attitudes, and practices of the population in the Jazan Region on microbiota composition and the main influencing factors. Furthermore, the use of a previously validated questionnaire in a similar context enhances the validity and reliability of the results. Moreover, differences in socioeconomic status might affect the level of knowledge of the microbiota of the participants. Finally, the large sample size of over 1,100 participants contributes to getting representative results. Despite its strengths, this study has limitations. First, the use of nonrandomized convenience sampling may introduce selection bias. Causality between the examined variables could not be established due to the cross-sectional nature of the study. Furthermore, the study relied on self-reported data, which may be subject to recall bias or social desirability bias. To address this limitation, future research could consider incorporating objective measures or conducting qualitative interviews to gain a more comprehensive understanding of participants‘ perspectives. While the use of a validated questionnaire increases the study‘s validity, the study‘s reliance on a single instrument may limit the scope of the information collected. Combining different research methods, such as surveys, interviews, or focus groups, can provide a more holistic view of participants‘ knowledge, attitudes, and practices toward microbiota and probiotics.

There is a need for increased education and awareness-raising efforts to correct misconceptions about microbiota and probiotics. Develop public health campaigns and educational programs to provide accurate and up-to-date information on the potential benefits of probiotics and the risks associated with the overuse of antibiotics, targeting the general population. HCPs are critical in promoting knowledge and understanding of microbiota and probiotics. HCPs should implement continuing education programs to improve their knowledge of microbiota, enabling them to provide accurate information and guidance to patients. In addition, it is important to develop interventions that target specific age groups, such as adolescents and older adults, to address the knowledge gaps identified in this study. Considering their access to information sources such as the internet and social media, we can tailor targeted educational materials and campaigns to these age groups‘ specific needs and preferences. Furthermore, it is important to enhance the general population‘s awareness and understanding of probiotics. Information about the health benefits of probiotics should be readily available through various channels, including healthcare facilities, schools, community centers, and online platforms. Finally, healthcare providers should promote appropriate antibiotic use by emphasizing the importance of completing the full course of therapy and discouraging using antibiotics without a medical prescription. Public awareness campaigns can help raise awareness about the risks of antibiotic misuse and the development of antibiotic resistance. By implementing these future recommendations, it is possible to improve the general population‘s knowledge, attitudes, and practices regarding microbiota and probiotics, ultimately promoting better health outcomes and preventing associated health problems.

## Conclusion

In conclusion, the survey reveals a general lack of awareness and understanding of probiotics. With most participants having poor knowledge of probiotics and a natural attitude toward antibiotics and probiotics, there is a need for additional education and awareness-raising about the importance of maintaining a healthy microbiota and the role that probiotics can play in achieving this. This could include public health campaigns and HCP education that gives accurate and up-to-date information on the different types of probiotics available, how they work, and the possible health advantages they may provide.

## List of abbreviations

GIT, gastrointestinal tract; IBD, inflammatory bowel disease; HCPs, healthcare professionals; SR, Saudi riyal; UAE, United Arab Emirates; MLR, multiple logistic regression; *P*, probability value.
